# Role of Lymphadenectomy During Interval Debulking Surgery Performed After Neoadjuvant Chemotherapy in Patients With Advanced Ovarian Cancer

**DOI:** 10.3389/fonc.2021.646135

**Published:** 2021-03-26

**Authors:** Minjun He, Yuerong Lai, Hongyu Peng, Chongjie Tong

**Affiliations:** ^1^ Department of Gynecologic Oncology, Sun Yat-sen University Cancer Centre, Guangzhou, China; ^2^ State Key Laboratory of Oncology in South China, Collaborative Innovation Centre for Cancer Medicine, Guangzhou, China

**Keywords:** lymphadenectomy, neoadjuvant chemotherapy, interval debulking surgery, advanced ovarian cancer, prognosis

## Abstract

**Objective:**

The role of lymphadenectomy in interval debulking surgery (IDS) performed after neoadjuvant chemotherapy (NACT) in advanced ovarian cancer remains unclear. We aimed to investigate the clinical significance of lymphadenectomy in IDS.

**Methods:**

We retrospectively reviewed and analyzed the data of patients with advanced ovarian cancer who underwent NACT followed by IDS.

**Results:**

In 303 patients receiving NACT-IDS, lymphadenectomy was performed in 127 (41.9%) patients. One hundred and sixty-three (53.8%) patients achieved no gross residual disease (NGRD), and 69 (22.8%) had residual disease < 1 cm, whereas 71 (23.4%) had residual disease ≥ 1cm. No significant difference in progression-free survival (PFS) and overall survival (OS) was observed between the lymphadenectomy group and the no lymphadenectomy group in patients with NGRD, residual disease < 1 cm, and residual disease ≥ 1 cm, respectively. The proportions of pelvic, para-aortic and distant lymph node recurrence were 7.9% (10/127), 4.7% (6/127) and 5.5% (7/127) in the lymphadenectomy group, compared with 5.7% (10/176, P = 0.448), 4.5% (8/176, P = 0.942) and 5.1% (9/176, P = 0.878), respectively, in no lymphadenectomy group. Multivariate analysis identified residual disease ≥ 1 cm [hazard ratios (HR), 4.094; P = 0.008] and elevated CA125 levels after 3 cycles of adjuvant chemotherapy (HR, 2.883; P = 0.004) were negative predictors for OS.

**Conclusion:**

Lymphadenectomy may have no therapeutic value in patients with advanced ovarian cancer underwent NACT-IDS. Our findings may help to better the therapeutic strategy for advanced ovarian cancer. More clinical trials are warranted to further clarify the real role of lymphadenectomy in IDS.

## Introduction

Ovarian cancer ranks the second leading cause of cancer-related death in women with gynecologic malignancies, with an estimated 313,959 new cases and 207,252 deaths in 2020 worldwide (1). In China, there were approximately 55,342 new cases of ovarian cancer and 37,519 ovarian cancer-related deaths in 2020 (1). Most women present with advanced disease at diagnosis. Approximately 75% of patients with advanced ovarian cancer will eventually relapse with the 5-year overall survival (OS) rate less than 25% (2).

For decades, the traditional treatment for advanced ovarian cancer is primary debulking surgery (PDS) followed by adjuvant chemotherapy. The goal of PDS is to remove the primary tumor and metastatic disease as much as possible. PDS typically includes the performance of a total abdominal hysterectomy (TAH) and bilateral salpingo-oophorectomy (BSO), a complete omentectomy (OM) and resection of any metastatic disease from the peritoneal surfaces, and an extensive resection of upper abdominal metastasis in some cases. Whether systematic lymphadenectomy should be part of maximal debulking surgery is still unclear. Several retrospective studies have suggested a significant survival benefit of systematic lymphadenectomy in patients undergoing cytoreduction (3–5). However, randomized controlled trials failed to demonstrate that systematic lymphadenectomy improved OS in women with optimally debulked ovarian cancer (6–8).

Recently, a series of trials demonstrated that neoadjuvant chemotherapy followed by interval debulking surgery (NACT-IDS) was non-inferior to PDS in progression-free survival (PFS) and OS and resulted in a lower incidence of treatment-related morbidity and mortality (9–12). Thus, NACT-IDS has been suggested as an alternative treatment for advanced ovarian cancer, especially for patients who are poor surgical candidates or have unresectable disease. Complete resection of all macroscopic disease remains the target regardless of PDS or IDS (13). Nevertheless, lymphadenectomy is not considered as a standard debulking procedure for IDS (9–11, 14). To date, there have been limited data on the role of lymphadenectomy in IDS performed after NACT in advanced ovarian cancer (15–17).

Since the 1990s, NACT-IDS has been performed in patients with advanced ovarian cancer in China. Therefore, in this paper, we analyzed patients with advanced ovarian cancer treated with NACT-IDS in our institution and aimed to investigate the role of lymphadenectomy in IDS.

## Materials and Methods

### Inclusion Criteria

After obtaining Institutional Review Broad approval, we performed a retrospective review of the clinical records of patients with epithelial ovarian cancer at Sun Yat-sen University Cancer Center between 2000 and 2014. Patients with stage III-IV disease who received NACT-IDS were eligible. The clinical data regarding patient demographics, surgical records, pathologic characteristics, treatment, follow-up, and vital status were extracted from the records. Patients who did not undergo IDS due to disease progression during NACT and who had PDS therapy as an initial treatment were excluded from the study. All methods were carried out in accordance with the approved guidelines of our institute.

### Treatment

In our department, NACT-IDS therapy is performed in patients with bulky stage III/IV disease who are not eligible surgical candidates. The majority of patients had histologic confirmation by core biopsy or diagnostic laparoscopy prior to NACT. NACT mainly included 2–3 cycles of first-line paclitaxel/docetaxel plus carboplatin/cisplatin. A combination of cyclophosphamide and bleomycin plus carboplatin was also used prior to 2003 when paclitaxel was not recommended as the first-line chemotherapy agent for the disease in our department.

NACT was followed by IDS unless the disease had progressed. Surgical procedures for IDS included TAH, BSO, and OM with or without the resection of various organs (e.g., bowel resection, diaphragm resection, or peritonectomy) to achieve optimal debulking. The performance of pelvic lymphadenectomy with or without para-aortic lymphadenectomy was left to the discretion of the surgeon. Lymph node sampling would be performed in patients with suspected/enlarged lymph nodes which was revealed in preoperative or intraoperative evaluation. In most instances, resection of enlarged lymph nodes rather than lymphadenectomy was performed in patients who did not gain optimal debulking. After IDS, 4–6 cycles of chemotherapy were planned for patients using the same regimen. However, the therapeutic strategy was altered if disease progression occurred.

### Statistical Analysis

Statistical analysis was performed using the SPSS 16.0 software (SPSS Inc., Chicago, IL, USA). The categorical variables were compared using the chi-square test. Survival time was calculated from the date of diagnosis. PFS was censored at the date of first recurrence or death or the date of the last contact for living patients without recurrent disease. OS was censored at the date of death or the date of the last contact for living patients. Survival curves were calculated using the Kaplan-Meier method and compared using the log-rank test. Multivariate analysis was performed using the Cox proportional hazards model to identify the prognostic factors that are independently associated with survival. Effects were expressed as hazard ratios (HR) with 95% confidence interval (CI). Statistical significance was defined as P < 0.05.

The key raw data have been recorded at Sun Yat-sen University Cancer Center for future reference (number RDDA2017000304).

## Results

### Patient Characteristics

Between January 2000 and 2014, a total of 761 patients with advanced epithelial ovarian cancer were identified, and 303 patients underwent NACT-IDS who were enrolled in the current study. The majority of patients (292/303, 96.4%) were judged inoperable for primary surgery by our center, and the remaining patients (11/303, 3.6%) were considered inoperable at the first surgery in other centers. The burden of the disease known at first diagnosis were 82.2% by CT or MRI or PET-CT, 14.2% by ultrasound, and 3.6% by laparoscopy. In these 303 patients, 127 (41.9%) patients had lymphadenectomy and 176 (58.1%) patients had no lymphadenectomy. In preoperative or intraoperative evaluation, 111 (36.6%) of 303 patients had suspected/enlarged lymph nodes, and 192 (63.4%) patients did not. The patient characteristics and adjuvant treatments according to lymphadenectomy were summarized in **Table 1**. The median follow-up time was 38.6 months (range, 1.7–177.9). The majority of patients (282/303, 93.0%) were diagnosed as stage IIIC-IV. NACT with a regimen of paclitaxel/docetaxel plus carboplatin/cisplatin was administered to 71.6% of the patients, and a regimen of cyclophosphamide and bleomycin plus carboplatin was administered to 20.5% of patients. Twenty-four (7.9%) patients underwent NACT with other regimens. The median number of NACT cycles was 2 (range, 1–6). Postoperative adjuvant chemotherapy with the same regimen was delivered to 84.2% of the patients, including 1–4 cycles in 25.4%, 5–6 cycles in 37.3%, and 7–8 cycles in 21.5%. The therapeutic strategy was changed for 48 (15.8%) patients who experienced disease progression during postoperative chemotherapy.

**Table 1:** Patient characteristics.

Characteristics	Overall N (%)	Lymphadenectomy Group N (%)	No-lymphadenectomy Group N (%)	P-value
All cases	303	127	176	NA
Age (year), median (range)	54 (23–75)	53 (32-74)	44.5 (23-75)	0.358
FIGO stage				0.191
IIIB	21 (6.9)	11 (8.7)	10 (5.7)	
IIIC	201 (66.3)	77 (60.6)	124 (70.5)	
IV	81 (26.7)	39 (30.7)	42 (23.9)	
Histology				0.451
Serous	187 (61.7)	77 (60.6)	110 (62.5)	
Mucinous	8 (2.6)	2 (1.6)	6 (3.4)	
Endometrioid	2 (0.7)	0 (0.0)	2 (1.1)	
Clear cell	5 (1.7)	2 (1.6)	3 (1.7)	
Mixed	2 (0.7)	0 (0.0)	2 (1.1)	
Adenocarcinoma, NOS	99 (32.7)	46 (36.2)	53 (30.1)	
Tumor grade				0.315
Grade 1	5 (1.7)	1 (0.7)	4 (2.3)	
Grade 2	21 (6.9)	8 (6.3)	13 (7.4)	
Grade 3	221 (72.9)	107 (84.3)	114 (64.7)	
Missing data	56 (18.5)	11 (8.7)	45 (25.6)	
NACT				0.000
Regimen				
TC/TP	217 (71.6)	106 (83.5)	111 (63.1)	
CBP	62 (20.5)	10 (7.9)	52 (29.5)	
Other regimens	24 (7.9)	11 (8.7)	13 (7.4)	
The number of cycles				0.028
Median (range)	2 (1-6)	2 (1-6)	2 (1-6)	
1–2	197 (65.0)	72 (56.7)	125 (71.0)	
3–4	90 (29.7)	48 (37.8)	42 (23.9)	
5–6	16 (5.3)	7 (5.5)	9 (5.1)	
Adjuvant chemotherapy				
The number of cycles				0.022
1–4	77 (25.4)	30 (23.6)	47 (26.7)	
5–6	113 (37.3)	59 (46.5)	54 (30.7)	
7–8	65 (21.5)	19 (15.0)	46 (26.1)	
Change of regimen for disease progression	48 (15.8)	19 (14.9)	29 (16.5)	
CA125 level prior to NACT (U/ml) Median (range)	1292 (10.1–47422.0)	1233.0 (10.1–31521.0)	1320.5 (13.4–47422.0)	0.457
Normalization of CA125 levels after 3 cycles of adjuvant chemotherapy				0.373
Yes	174 (57.4)	73 (57.5)	90 (51.1)	
No	111 (36.6)	31 (24.3)	50 (28.4)	
Missing data	18 (5.9)	23 (18.1)	36 (20.5)	

NOS, Not otherwise specified; NACT, neoadjuvant chemotherapy; TC/TP, Paclitaxel/docetaxel plus carboplatin/cisplatin; CBP, Cyclophosphamide, bleomycin plus carboplatin.

### Surgical Procedures

The surgical procedures included TAH + BSO + OM in 140 (140/303, 46.2%) patients, pelvic lymphadenectomy in 127 patients (127/303, 41.9%), para-aortic lymphadenectomy in 19 patients (19/303, 6.3%), and resection of other organs in 65 (65/303, 21.5%) patients. The IDS surgical performance and pathologic results were summarized in **Table 2**. There were 140 patients who had lymphadenectomy or lymph node sampling, including 78 (55.7%, 78/140) patients with lymph node metastasis and 62 (44.3%, 62/140) with lymph node negative. The median number of resected nodes from pelvic lymphadenectomy and para-aortic lymphadenectomy was 20 (range, 8–59) and 10 (range, 8–24), respectively. Regarding the status of residual disease, 163 (163/303, 53.8%) patients achieved no gross residual disease (NGRD), and 69 (69/303, 22.8%) patients had optimal debulking (residual disease < 1 cm). However, 71 (71/303, 23.4%) patients had residual disease ≥ 1 cm.

**Table 2:** Surgical procedures applied in patients.

	Patient number (%) (N=303)
Surgical procedure	
TAH + BSO + OM	140 (46.2)
TAH + BSO + OM + Pelvic lymphadenectomy/LNS	62 (20.5)
TAH + BSO + OM + Pelvic lymphadenectomy/LNS + Para-aortic lymphadenectomy/LNS	36 (11.9)
TAH + BSO + OM + resection of other organs	23 (7.6)
TAH + BSO + OM + Pelvic/para-aortic lymphadenectomy/LNS + resection of other organs	42 (13.9)
Lymph node resection	
Pelvic lymphadenectomy	127 (41.9)
Pelvic LNS	13 (4.3)
Para-aortic lymphadenectomy	19 (6.3)
Para-aortic LNS	46 (15.2)
Number of resected lymph node	
Pelvic lymphadenectomy, median (range)	20 (8–59)
Pelvic LNS, median (range)	3 (1–7)
Para-aortic lymphadenectomy, median (range)	10 (8–24)
Para-aortic LNS, median (range)	3 (1–7)
Residual disease	
NGRD	163 (53.8)
Optimal (residual disease < 1cm)	69 (22.8)
Suboptimal (residual disease ≥ 1cm)	71 (23.4)

LNS, lymph node sampling; NGRD, no gross residual disease.

### The Outcomes According to the Performance of Lymphadenectomy

**Figure 1:**
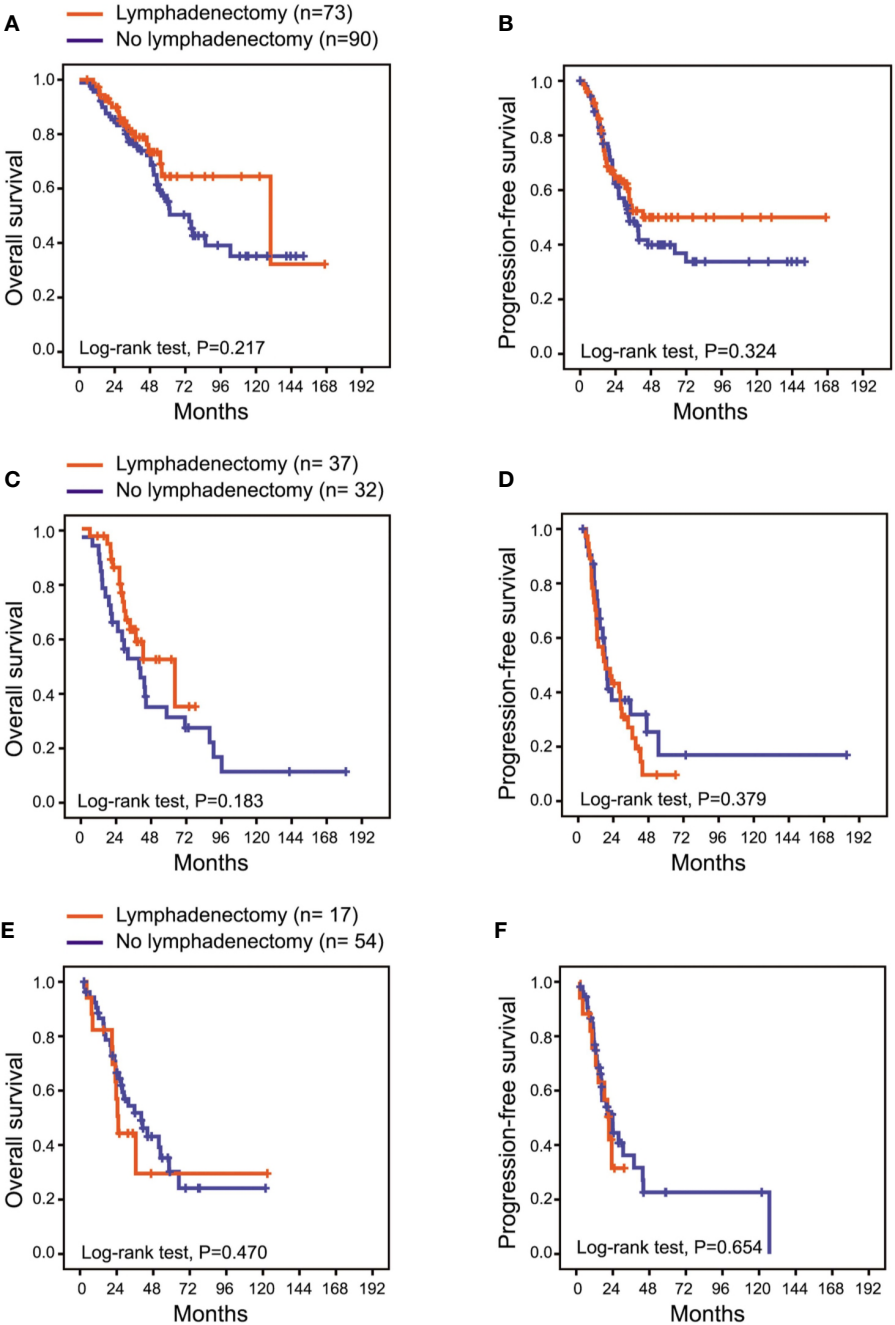
Overall survival and progression-free survival according to three different residual disease statuses. **(A, B)**, patients with no gross residual disease; **(C, D)** patients with residual disease < 1 cm; **(E, F)** patients with residual disease ≥ 1cm.

Given that the surgeons commonly performed a lymphadenectomy according to whether the debulking surgery was optimal, we analyzed the influence of lymphadenectomy on patient prognosis according to the residual disease status. In the 163 patients with NGRD, 73 (73/163, 44.8%) patients had lymphadenectomy, and 90 (90/163, 55.2%) patients did not. The 3-year PFS and 5-year OS rates were 52.4% and 64.5%, respectively, in the lymphadenectomy group and 48.6% and 55.2%, respectively, in the no lymphadenectomy group. Among the 69 patients with optimal debulking, 37 (37/69, 53.6%) patients had lymphadenectomy, and 32 (32/69, 46.4%) patients did not. The 3-year PFS and 5-year OS rates were 27.0% and 52.0%, respectively, in the lymphadenectomy group and 31.8% and 34.5%, respectively, in the no lymphadenectomy group. In the 71 patients with residual disease ≥ 1 cm, only 23.9% (17/71) of the patients underwent lymphadenectomy. The 3-year PFS and 5-year OS rates were 31.5% and 29.6%, respectively, in the lymphadenectomy group and 36.2% and 30.2%, respectively, in the no lymphadenectomy group. No significant differences in PFS as well as OS were observed between the lymphadenectomy group and the no lymphadenectomy group in patients with NGRD, optimal debulking, and suboptimal debulking, respectively (**Table 3**). **Figure 1** presented the PFS and OS for different residual disease statuses in the lymphadenectomy group and in the no lymphadenectomy group.

**Table 3:** Progression-free survival and overall survival according to the performance of lymphadenectomy.

	3-year PFS			5-year OS		
	Lymphadenectomy			Lymphadenectomy		
	Yes	No	P value	Yes	No	P value
No gross residual disease	52.4%	48.6%	0.324	64.5%	55.2%	0.217
Residual disease < 1 cm	27.0%	31.8%	0.379	52.0%	34.5%	0.183
Residual disease ≥ 1 cm	31.5%	36.2%	0.654	29.6%	30.2%	0.470

### Post-Operative Major Complications

Post-operative major complications were summarized in **Table 4**. The incidence of major complications in lymphadenectomy group was higher than that in no-lymphadenectomy group (11.8% vs 5.7%, P=0.039). Fever was the most common post-operative complications both in lymphadenectomy group (4/127, 3.1%) and no-lymphadenectomy group (7/176, 4.0%). The second most common post-operative complications were lymphatic cyst (3/127, 1.8%) in lymphadenectomy group and infection (3/176, 1.7%) in no-lymphadenectomy group.

**Table 4:** Post-operative major complications.

Complication	Lymphadenectomy Group (n = 127) N (%)	No-lymphadenectomy Group (n = 176) N (%)	P-value
Complication			0.039
Yes	15 (11.8)	10 (5.7)	
No	112 (88.2)	166 (94.3)	
The specific complications			
Fever with body temperature>38°C	4 (3.1)	7 (4.0)	
Lymphatic cyst	3 (1.8)	0 (0.0)	
Infection treated with antibiotics	1 (0.8)	3 (1.7)	
Lymphedema	1 (0.8)	0 (0.0)	
Peripheral sensory neurologic event	1 (0.8)	0 (0.0)	
Ileus	1 (0.8)	0 (0.0)	
Fistula	1 (0.8)	0 (0.0)	

### Recurrence Characteristics Based on the Performance of Lymphadenectomy

In our cohort, the recurrent disease were diagnosed by elevated CA125 levels (55/303, 32%), CT/MRI (80/303, 46.5%), PET/CT (35/303, 20.3%), and surgery (2/303, 1.2%). Most patients recurred first with peritoneal dissemination or distant metastasis, including 51 patients (51/127, 40.2%) in the lymphadenectomy group and 79 (79/176, 44.9%) in the no lymphadenectomy group. In the no lymphadenectomy group, only 4.5% of the patients (8/176) had the first site of recurrence confined to the lymph node, which was comparable with the rate (6/127, 4.7%, chi square test, P = 0.942) in the lymphadenectomy group. Furthermore, 5.7% (10/176) of patients in the no lymphadenectomy group and 7.9% (10/127) of patients in the lymphadenectomy group showed first recurrence *via* peritoneal dissemination simultaneously with lymph node metastasis.

The proportions of pelvic, para-aortic and distant lymph node recurrence were 7.9% (10/127), 4.7% (6/127) and 5.5% (7/127) in the lymphadenectomy group, respectively, compared with 5.7% (10/176, P = 0.448), 4.5% (8/176, P = 0.942) and 5.1% (9/176, P = 0.878), respectively, in no lymphadenectomy group.

### Multivariate Cox Regression Model Analysis

Multivariate Cox regression model analyses of PFS and OS were presented in **Table 5**. Multivariate analysis of PFS identified FIGO stage IV (HR, 1.843; 95% CI, 1.058–3.211; P = 0.031) as a negative prognostic factor. Moreover, residual disease < 1 cm (HR, 2.093; 95% CI, 1.218–3.595; P = 0.007) and ≥ 1 cm (HR, 2.568; 95% CI, 1.172–5.625; P = 0.018) were associated with poor PFS compared with NGRD. Multivariate analysis for OS revealed that residual disease ≥ 1 cm (HR, 4.094; 95% CI, 1.456–11.514; P = 0.008) and elevated CA125 levels after 3 cycles of adjuvant chemotherapy (HR, 2.883; 95% CI, 1.409–5.902; P = 0.004) were significantly correlated with worse OS rates. In addition, no statistical significance in lymph node metastasis was noted for both OS and PFS.

**Table 5:** Multivariable Cox regression model analyses for progression-free and overall survival.

Variable	PFS		OS	
	HR (95% CI)	P value	HR (95% CI)	P value
**Age**				
≤60 years	1		1	
>60 years	1.245 (0.600–2.583)	0.556	1.763 (0.656–4.734)	0.261
**FIGO stage**				
Stage III	1		1	
Stage IV	1.843 (1.058–3.211)	0.031	1.685 (0.792–3.585)	0.176
**Residual disease**				
No gross residual disease	1		1	
Optimal (< 1 cm)	2.093 (1.218–3.595)	0.007	1.510 (0.695–3.282)	0.298
Suboptimal (≥ 1 cm)	2.568 (1.172–5.625)	0.018	4.094 (1.456–11.514)	0.008
**Histologic types**				
Serous	1		1	
Non-serous	1.216 (0.718–2.060)	0.466	0.688 (0.324–1.459)	0.330
**Tumor grade**				
Grade 1	1		1	
Grade 2	1.867 (0.219–15.907)	0.568	0.321 (0.031–3.340)	0.341
Grade 3	0.611 (0.080–4.668)	0.635	0.187 (0.022–1.556)	0.121
**Lymph node metastasis** ^#^				
Positive	1		1	
Negative	0.758 (0.429–1.339)	0.340	1.491 (0.684–3.249)	0.315
**Normalization of CA125 levels after 3 cycles of adjuvant chemotherapy**				
Yes	1		1	
No	1.610 (0.957–2.709)	0.073	2.883 (1.409–5.902)	0.004

^#^Only 140 patients who underwent lymphadenectomy or lymph node sampling were included.

## Discussion

To date, the role of lymphadenectomy in patients with advanced disease remains controversial (3–7). The dispute has focused on whether lymphadenectomy should be performed for surgical staging as well as on its therapeutic benefit. Two previous randomized controlled trials demonstrated that patients who underwent systematic lymphadenectomy during PDS had a higher proportion of lymph node metastasis compared with patients who had no lymphadenectomy, which made the apparent early stage ovarian cancer patients upstaged due to occult lymph node metastasis (6, 7). These data favored lymphadenectomy for the purpose of surgical staging in early stage ovarian cancer. However, it could be certain that the performance of lymphadenectomy does not influence the accurate assessment of staging in IDS, given that IDS is mostly delivered to patients with stage IIIC-IV ovarian cancer.

The therapeutic value of lymphadenectomy continues to be debated in both PDS and IDS. There is no doubt that complete resection achieving no gross residual disease, by either PDS or IDS, is the most important predictor for outcome in patients with advanced ovarian cancer (9). The present study also demonstrated that no gross residual disease in IDS was an independent prognostic factor for both PFS and OS. Thus, the removal of any bulky lymph node must be performed to obtain NGRD from the position of maximal debulking. Nevertheless, the core issue is whether lymphadenectomy should be performed in optimal debulking and complete resection to remove occult metastatic lymph nodes. Most randomized controlled trials did not demonstrate the therapeutic benefit of systematic lymphadenectomy during PDS in women with optimally debulked ovarian cancer (6–8). However, the therapeutic value of lymphadenectomy during IDS remains controversial to date (15–17). Fagotti et al. (15) disclosed that systematic pelvic lymphadenectomy and para-aortic lymphadenectomy during IDS had no value in improving PFS and OS in patients with advanced ovarian cancer. Likewise, the data from Iwase et al. (16) indicated that systematic retroperitoneal lymphadenectomy during IDS could predict the outcome of patients with advanced ovarian cancer; however, it did not improve patient prognosis. In our study, due to selection bias in the performance of lymphadenectomy, which was commonly not done in patients who did not achieve optimal debulking, we analyzed the influence of lymphadenectomy on patient prognosis according to the residual disease status. In line with previous studies, we failed to identify a significant benefit of lymphadenectomy performance for PFS and OS in patients with no gross residual disease, optimal debulking, and suboptimal debulking, respectively. Nevertheless, other studies reported the opposing results. Bund et al. revealed that patients with initially inoperable advanced ovarian cancer and treated with NACT-IDS and systematic lymphadenectomy had significantly higher PFS regardless of node-positivity status compared to those who did not have systematic lymphadenectomy (18). Eoh et al. (17) demonstrated that PFS was significantly better in the patients with lymphadenectomy during IDS who were preoperatively negative lymphadenopathy, comparing with those undergone lymph node sampling. These findings suggested that systematic lymphadenectomy might have therapeutic value in patients with advanced ovarian cancer during IDS. It was reported that complete resection of occult metastatic lymph nodes might decrease the risk of chemoresistance (19, 20), which was considered by Eoh et al. as a possible explanation for their findings. In addition, some study showed that the diagnosis center might influence the prognosis of ovarian cancer patients submitted to NACT (21). It may to some extent explain why there were disputable results in the role of lymphadenectomy in patients with ovarian cancer during IDS. However, further investigation is warranted to identify its therapeutic benefit.

In the present study, the first recurrence site confined to the lymph nodes was only observed in 4.5% of the patients in the no lymphadenectomy group, which was comparable with the rate (4.7%, P=0.942) in the lymphadenectomy group. Regarding the characteristics of subsequent recurrence in our study, the procedure of lymphadenectomy did not seem to contribute to reduce the nodal relapse. Our findings were consistent with previous data. In the report from Iwase et al. (16), many patients recurred with peritoneal dissemination, and the rate of recurrence in the lymph nodes was comparable regardless of the performance of lymphadenectomy. Notably, among the patients who underwent lymphadenectomy, there was no difference in the outcome of patients with lymph node positive and negative disease. Currently, the prognostic value of lymph node metastasis in ovarian cancer remains unclear (4, 22–24). Previous investigations have shown that the influence of lymph node metastasis on prognosis decreased with the increase in residual disease during PDS (4, 24). In our data, lymph node metastasis was not an independent prognostic factor of outcome. Given that most of our patients (93%) exhibited stage IIIC-IV disease with bulky intraperitoneal implantation, we propose that the prognostic impact of complete resection with no gross residual disease might be more important than node metastasis, which was consistent with the observation in PDS.

To the best of our knowledge, this is the largest single-institution series of patients with advanced ovarian cancer who underwent IDS with or without lymphadenectomy. However, there were several limitations in our study due to its retrospective nature, and the results should be interpreted with caution. First, most of our patients did not have enough resected para-aortic lymph nodes, which may lead to underestimate of the therapeutic value of lymphadenectomy. In the study by Panici et al. (6), pelvic lymphadenectomy was considered appropriate when at least 25 nodes were removed, and para-aortic lymphadenectomy requires the removal of at least 15 lymph nodes. According to these criteria, the procedure could be defined as para-aortic lymphadenectomy in only 2.6% (8/303) of our patient population, and only 17.8% (54/303) of patients had appropriate pelvic lymphadenectomy. In our institute, lymphatic fatty tissue is removed en bloc and split according to region for pathologic examination. Then, lymph node separation is performed by the pathologist. Therefore, the number of lymph nodes that are noted in the pathologic report may be influenced by the time spent by the pathologist on performing the separation. This may be a plausible explanation for the insufficiency of resected lymph nodes in our study. However, the boundaries of the lymph node procedure are similar to those outlined in the study by Panici et al. (6). Second, the performance of lymphadenectomy was left to the discretion of the surgeon, which may result in bias. Third, the population enrolled in our study was heterogeneous and lack of data on BRCA gene status.

## Conclusion

In conclusion, the present study showed that lymphadenectomy may have no therapeutic value in patients with advanced ovarian cancer underwent NACT-IDS. Our findings may help to better the therapeutic strategy for advanced ovarian cancer. More clinical trials are warranted to further clarify the real role of lymphadenectomy in IDS.

## Data Availability Statement

The datasets presented in this article are not readily available because data from the Research Data repository (https://www.researchdata.org.cn/default.aspx) was used under license for the current study. The data can be accessed using identifier RDDA2017000304, but permission must be acquired. Requests to access the datasets should be directed to the corresponding author.

## Author Contributions

MH: Conception and design, and manuscript writing. YL: Collection and assembly of data, and manuscript revision. HP: Methodology and writing original draft preparation. CT: Conception and design, and manuscript revision. All authors contributed to the article and approved the submitted version.

## Conflict of Interest

The authors declare that the research was conducted in the absence of any commercial or financial relationships that could be construed as a potential conflict of interest.
